# Cinnamaldehyde impacts key cellular signaling pathways for induction of programmed cell death in high-grade and low-grade human glioma cells

**DOI:** 10.1186/s13104-025-07092-8

**Published:** 2025-01-20

**Authors:** Yoo Na Kim, Ketki Patil, S. Balakrishna Pai

**Affiliations:** Wallace H. Coulter Department of Biomedical Engineering, Georgia Institute of Technology and Emory University, 313 Ferst Drive, Atlanta, GA 30332 USA

**Keywords:** High-grade glioma, Low-grade glioma, Cinnamaldehyde, U251, H4, Apoptosis, Bcl-2, Reactive oxygen species, Multicaspase

## Abstract

**Objective:**

Primary tumors of the brain and a large percent of malignant brain tumors are gliomas. Gliomas comprise high-grade gliomas like glioblastoma multiforme (GBMs), many of which have mutation in the tumor suppressor p53 gene and low-grade gliomas (LGGs). LGGs can progress to GBMs due to various factors. The available treatment options for GBMs and LGGs include surgical resection, radiation and chemotherapy. The chemotherapeutic drug available in the clinic is temozolomide (TMZ). However, TMZ can cause damage to DNA if taken for prolonged period. This warrants the discovery of drugs that would potentially elicit less adverse side effects while maintaining anticancer activity. To this end, we evaluated the impact of cinnamaldehyde (CA), a single, purified component of the natural product cinnamon.

**Results:**

The elucidation of the mechanism of action revealed the impact of CA on reactive oxygen species (ROS) levels. Moreover, its effect on the extrinsic programmed cell death pathway resulted in the increase of apoptotic cell populations, invoking multicaspase. Notably, the cell survival/death pivotal molecule Bcl-2 was impacted. These effects were observed in both the types of brain tumor cells studied: GBMs, represented by U251 cells (p53 mutated cell line) and LGGs represented by H4 cells. Results from the current study suggest potential for CA as a therapeutic option as it is expected to have fewer adverse side effects due to it being a component of a natural product and possibly deter the progression of LGGs to GBMs.

**Supplementary Information:**

The online version contains supplementary material available at 10.1186/s13104-025-07092-8.

## Introduction

Glioblastoma is the most frequently diagnosed brain cancer accounting for 47.7% of all cases with an incidence rate of 3.21 per 100,000 population [1]. An estimated more than 10,000 people in the US will be diagnosed every year with glioblastoma with a five-year survival rate of 6.9% [2]. Transformation of the progenitors of glial cells or stem cells of the central neural system may lead to the formation of brain tumors [3]. Further, contribution by common and various progenitors of glial cells during brain tumor formation is characterized [4]. Among the brain tumors, a large percent of primary malignant brain tumors are characterized as gliomas [5]. Clinically, gliomas are divided into different grades (I to IV) ranging from low-grade to high-grade [6], and glioblastoma multiforme (GBM) is a grade IV tumor; this is the most aggressive form of tumor with various risk factors, difficult to track and treat with a median survival of about 15 months and with poor prognosis [7–9]. With respect to gliomas, studies have been carried out in understanding the signaling pathways and molecules which could lead to non-responsiveness to current therapeutic treatments [10]. Also, alterations in the tumor suppressor p53 is often observed in brain tumors. Primary GBMs as well as secondary GBMs exhibit changes in the tumor suppressor p53 and is considered as one among the molecular markers [11, 12].

Currently available treatment options for GBMs include surgery, radiation and chemotherapy. Often, the chemotherapeutic drug used in the clinic is temozolomide (TMZ). Also, combination of TMZ and radiation is reported [13]. In older patients, treatment with TMZ did not improve the prognosis [14]. Moreover, it has been reported that tumors which recurred on TMZ treatment had hypermutations [15]. To achieve improved treatment options, efforts have also been directed towards bioengineering strategies in animal model systems [16]. To mitigate potential side effects associated with TMZ and for efficacious treatment of tumors of the brain, there is an urgent need for the development of more safe drugs.

With respect to LGGs, there are various tumor types and variations in histologic characteristics. Further, recently, molecular subtypes that can be identified in diffuse gliomas including immune evasion mechanisms that could operate in these cells are reported [17]. Notably, gliomas have been characterized and grouped based on the presence of certain markers reflecting on the pathogenesis of these tumors [18]. Current treatment employed for LGGs is safe resection, MRI monitoring in low-risk individuals; radiation and chemotherapy for high-risk patients [19, 20].

The U87 cell line with wild type p53, U251 with mutated p53 for high grade gliomas and H4 cell line for LGG are frequently utilized in the study and discovery of drugs against gliomas. Recently, molecular studies have been carried out with the GBM cell line U87MG (grade IV glioblastoma) and the nontumorigenic LGG cell line, H4 related to mTORC2 and the entities it interacts with to understand aggressiveness of high-grade gliomas [21]. Furthermore, detailed study comparing less aggressive glioma-like H4 cells and high-grade, highly aggressive glioma has been reported affecting cell signaling pathways controlling migration as well [22]. Although patients with LGG survive for a longer time than patients with GBM, the tumor can result in secondary GBM over time and targeted therapy for these tumors is being investigated [23]. Also, various factors leading to malignant transformation of low-grade gliomas is reported [24]. Moreover, by differential gene expression analysis, potential therapeutic target has been identified in LGG which could lead to malignant tumors [25]. Recently, the involvement of cancer-associated fibroblasts, epigenetics and its connection to the tumor stroma has been reported to possibly lead to targeted therapy along with immune therapy for LGG [26]. Improvement and novel treatment for patients with LGG considering tumor and constitutive genetics has also been suggested to be addressed [27]. Importantly, there is no curative option for low-grade gliomas as well, although the patients with LGG live for a long time. Recently, we have reported in detail the potential impact of CA, a highly purified ingredient of the natural product cinnamon, on U87 (wild-type p53) glioblastoma cells. Also, we have reported dose-dependent effect of CA on cell viability in GBM cells with p53 mutation (U251 cell line) and in H4 cells which are low-grade glioma cells [28]. In the present study, we report the elucidation of the molecular mechanism of action of CA on both the brain tumors, GBM with p53 mutation which is observed in majority of GBMs and LGGs, as there is a need to evaluate the cell entities affected to consider CA as a potential option for future treatment with the anticipation of less side effects.

## Materials and methods

### Cell lines, materials and cell toxicity assay

Commercially available U251 cell line (Sigma-Aldrich, MO, USA) and H4 Cell line (American Type Culture Collection, Manassas, VA, USA) were purchased and cultured as per the instructions of the supplier. Laboratory use of the cell lines were approved by the Biological Material Safeguards Committee of Georgia Institute of Technology. Cinnamaldehyde was purchased from Sigma-Aldrich (Rockville, MD, USA). Luminex Muse Cell analyzer and Muse assay kits were bought from EMD Millipore (Burlington, MA, USA). Stock solution of CA was prepared in DMSO. Dose-dependent inhibition of U251 and H4 cell lines was assessed by treating the cells with varying concentrations of CA and determining the cell viability using the Cell Counting Kit- 8 (Bimake, Houston, TX, USA) as described earlier [28].

### Flow cytometry assays

U251 cells at 62,000 cells per well were plated in 12-well plates. After 24 h, cells were either left untreated (control group) or treated in triplicate with CA and incubated for 72 h. After 72 h, media was collected, adhered cells were trypsinized and pooled together from triplicate treatments. The samples were then subjected to Luminex Muse assays as per manufacturer’s instructions. Flow cytometry assay kits used to assess key cell entities were: Oxidative Stress kit (MCH100111), Annexin V and Dead Cell Kit (MCH100105), MultiCaspase kit (MCH100109), and Bcl-2 kit (Bcl-2 Dual detection activation assay (MCH200105) and Mitopotential Assay kit (MCH100110). Molecular assays with the H4 cell line utilized similar number of cells and flow cytometry protocols as those employed for the U251 cell line.

### Statistical analysis

Unpaired two-tailed t test with Welch’s correction, for ROS, statistical significance at *P* < 0.05 and Sidak’s one-way ANOVA with Dunnett’s multiple comparison test was calculated and mentioned in the legend section to figures.

## Results

### Reactive oxygen species levels were elevated in U251 and H4 cells treated with CA

**Fig. 1:**
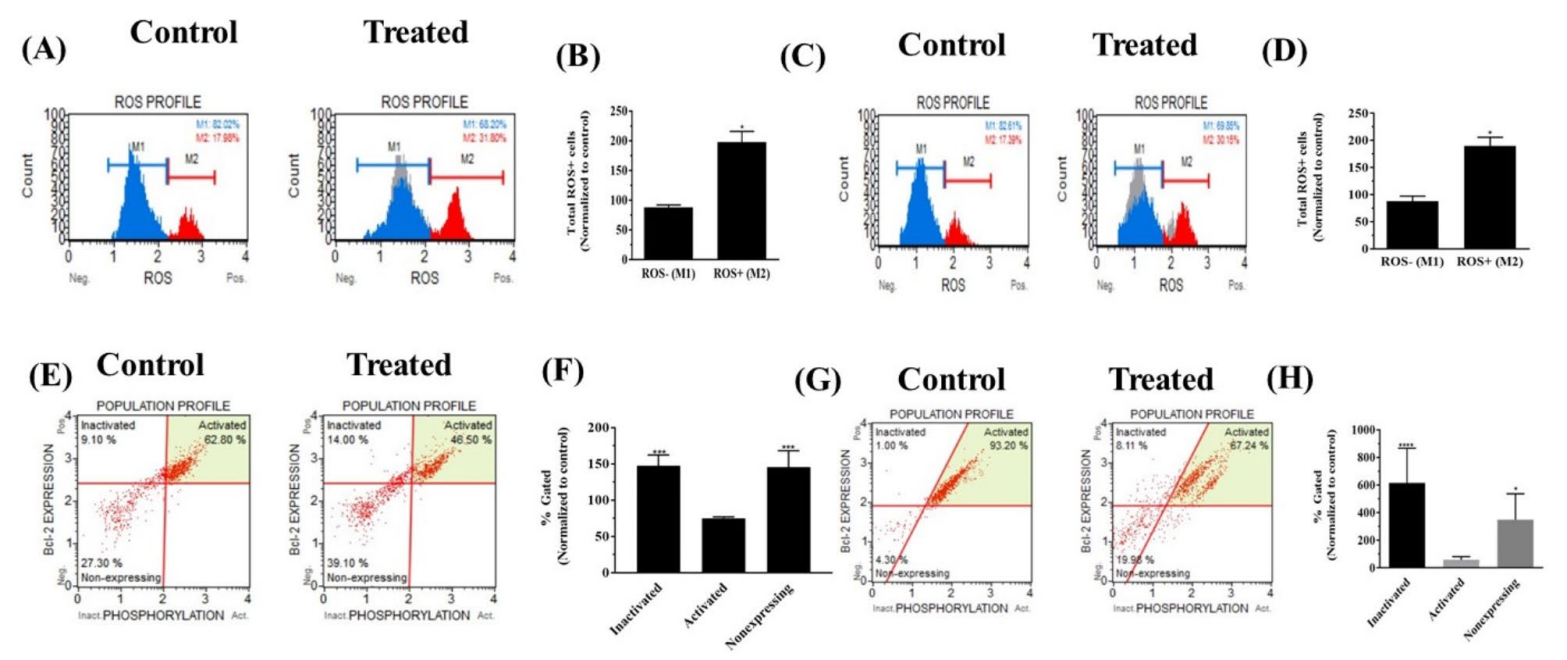
Profile of reactive oxygen levels on treatment with CA in H4 and U251 cells as well as impact on Bcl-2. **(A)** Representative ROS profile obtained for H4 cells untreated or treated with CA. **(B)** On normalizing to the control group there was a significant increase in the ROS + cell population. **(C)** Representative ROS profile obtained for U251 cells untreated or treated with CA. (**D**) On normalizing to the control group there was a significant increase in the ROS + cell population. For both H4 and U251, unpaired two-tailed t test with Welch’s correction was conducted and p value of 0.0115 for H4 and 0.0106 for U251. **(E)** Representative scatter plots depicting the Bcl-2 cell populations with inactivated, activated and non-expressing cells in H4 cells on treatment with CA. **(F)** A statistically significant increase in the inactivated Bcl-2 molecules as well as statistically significant increase in the non-expressing cell population was observed by two-way ANOVA with Sidak’s multiple comparisons test with a p value of 0.0007 for the inactivated population and p value of 0.0010 for non-expressing population. **(G)** Representative scatter plots of Bcl-2 levels in the U251 cells treated with CA and in the untreated control group. **(H)** Bar diagram depicting a statistically significant increase in the inactivated Bcl-2 molecules as well as statistically significant increase in the non-expressing cell population in U251 cells as determined by two-way ANOVA with Sidak’s multiple comparisons test with a p value of < 0.0001 for inactivated population and p value of 0.0265 for non-expressing population

Elucidation of the mechanism of action of CA in the GBM cell line, U251 and LGG cell line, H4 was performed by first analyzing the production of ROS using the Oxidative Stress kit (as in ‘Materials and Methods’). For this analysis, the cells were treated with the IC_50_ concentration (80µM) of CA observed for H4 (as reported previously [28]) and the same concentration was used for U251 cells and incubated for 72 h. Increase in ROS levels were observed in each of the cell lines. The assay was repeated three independent times and representative profile obtained for the treated samples in comparison to the profile obtained for the control samples for H4 cells is shown (Fig. 1A). The values obtained for the treated samples on normalizing to the control samples are shown, (Fig. 1B). For U251 cells, a similar increase in ROS levels was observed. Three independent assays were performed and representative profile obtained for the treated group in comparison with the profile obtained for the control group is shown, (Fig. 1C). The values obtained for U251 cells treated with CA normalized to the control group are shown, (Fig. 1D). As seen in (Fig. 1B) and (Fig. 1D) total increase in ROS + cells generated on CA treatment were statistically significant in each of the cell systems analyzed.

### CA treatment affects Bcl-2 levels in H4 and U251 cells

Bcl-2 is a pivotal molecule involved in the control of cell survival and cell death of various types of cancers. To investigate the effect of CA on Bcl-2 levels in the LGG cell line H4 and GBM cell line U251, a Bcl-2 flow cytometric assay was performed. Three independent experiments were performed for each cell line. Representative scatter plots of Bcl-2 levels in the H4 cells treated with CA and in the untreated control group are shown (Fig. 1E). As seen from the plots, the percentage of cell population with the inactivated Bcl-2 has increased in the CA treated group with concomitant decrease in the activated Bcl-2, also increase in the Bcl-2 non-expressing cells was observed, (Fig. 1F). A statistically significant increase in the inactivated Bcl-2 molecules as well as in the non-expressing cell population was observed as per ANOVA. Similarly, the levels of the key molecule Bcl-2 was monitored in the U251 cell line. Representative scatter plots of Bcl-2 level in the U251 cells treated with CA and in the untreated control group are shown (Fig. 1G). In the CA treated group, the percentage of cell population with the inactivated Bcl-2 has increased and a concomitant decrease in the activated Bcl-2 molecules and increase in the Bcl-2 non-expressing cells was observed. In the U251 cells as well, a statistically significant increase in the inactivated Bcl-2 molecules as well as statistically significant increase in the non-expressing cell population was observed (Fig. 1H).

### Impact on the programmed cell death pathway by CA in the LGG (H4) and GBM (U251) cells

**Fig. 2:**
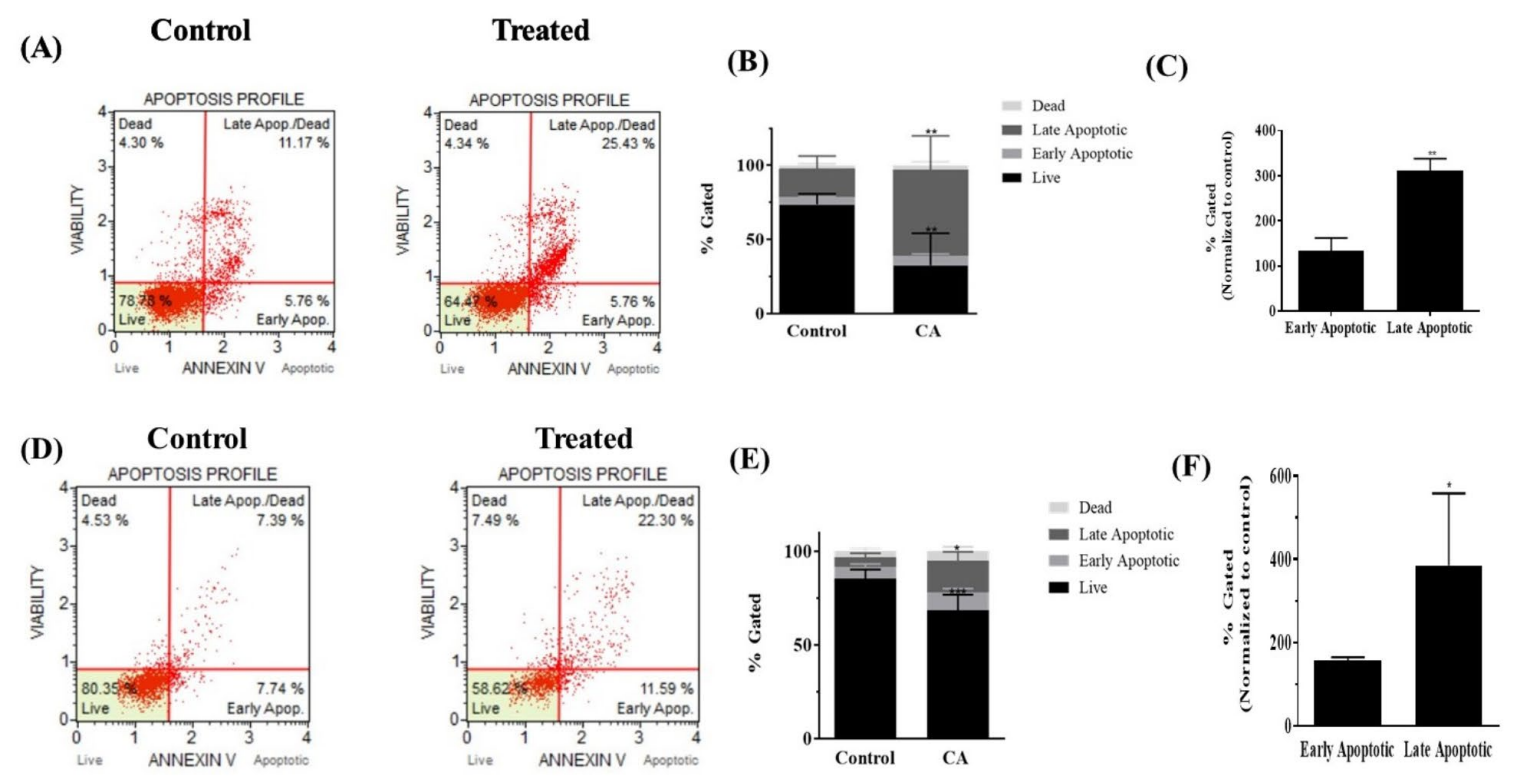
Analysis of apoptotic cell populations in H4 and U251 cells treated with CA. To generate the data, three independent experiments were performed. **(A)** Representative scatter plot of apoptotic cell populations detected using Annexin V and Dead Cell Kit in H4 cells treated with 80 µM of CA for 72 h. **(B)** Percent gated profile showing the different cell populations from untreated and treated cells. A significant change was observed in all the different cell populations of the treated groups as per two-way ANOVA with Sidak’s multiple comparisons test. Significant decrease in live cells (p value = 0.0025) and significant increase in late apoptosis (p value = 0.0041) was observed. **(C)** On normalizing to the control group, both early and late apoptotic cell populations are represented. One-way ANOVA with Dunnett’s multiple comparisons test showed significant increase in late apoptosis (p value = 0.0086). **(D)** Representative scatter plots of apoptotic cell populations detected using Annexin V and Dead Cell Kit in U251 cells treated with 80 µM of CA for 72 h. **(E)** The percent gated profile of the cell populations from untreated and treated U251 cells is shown. All the different cell populations showed a significant change in the treated samples by two-way ANOVA with Sidak’s multiple comparisons test. A significant decrease in live population (p value = 0.0005) and a significant increase in late apoptosis (p value = 0.0119) was observed. **(F)** The early and late apoptotic cell populations in the treated U251 cells are represented on normalizing to the control group. One-way ANOVA with Dunnett’s multiple comparisons test shows a significant increase in late apoptosis (p value = 0.0230)

On observing significant increase in ROS levels in both the types of brain tumor cells by CA, we proceeded to analyze its effect on the extrinsic programmed cell death pathway by performing flow cytometric assay using the Luminex Muse Annexin V and Dead Cell assay. H4 and U251 cells were treated with 80 µM of CA and were incubated for 72 h. The experiment was repeated three independent times and representative scatter plots of the control group and treated group of H4 cells are shown in (Fig. 2A) and the percent gated profile of each of the cell population in the untreated and treated group is shown (Fig. 2B). Total number of apoptotic cells increased significantly in treated samples. The percent of early and late apoptotic cell population normalized to the control population from three independent experiments is represented, (Fig. 2C). Also, for U251 cells, the experiments were repeated three independent times and the representative scatter plots of the control sample and treated sample are shown, (Fig. 2D). The percent gated profile of each of the cell population in the untreated and treated sample is shown, (Fig. 2E). In U251 cells as well, the total number of apoptotic cells increased significantly in the treated samples. With respect to the apoptotic cells in the U251 sample, the percent of early and late apoptotic cell population normalized to the control population from three independent experiments is represented, (Fig. 2F).

### Multicaspase are invoked by CA in H4 and U251 cells

**Fig. 3:**
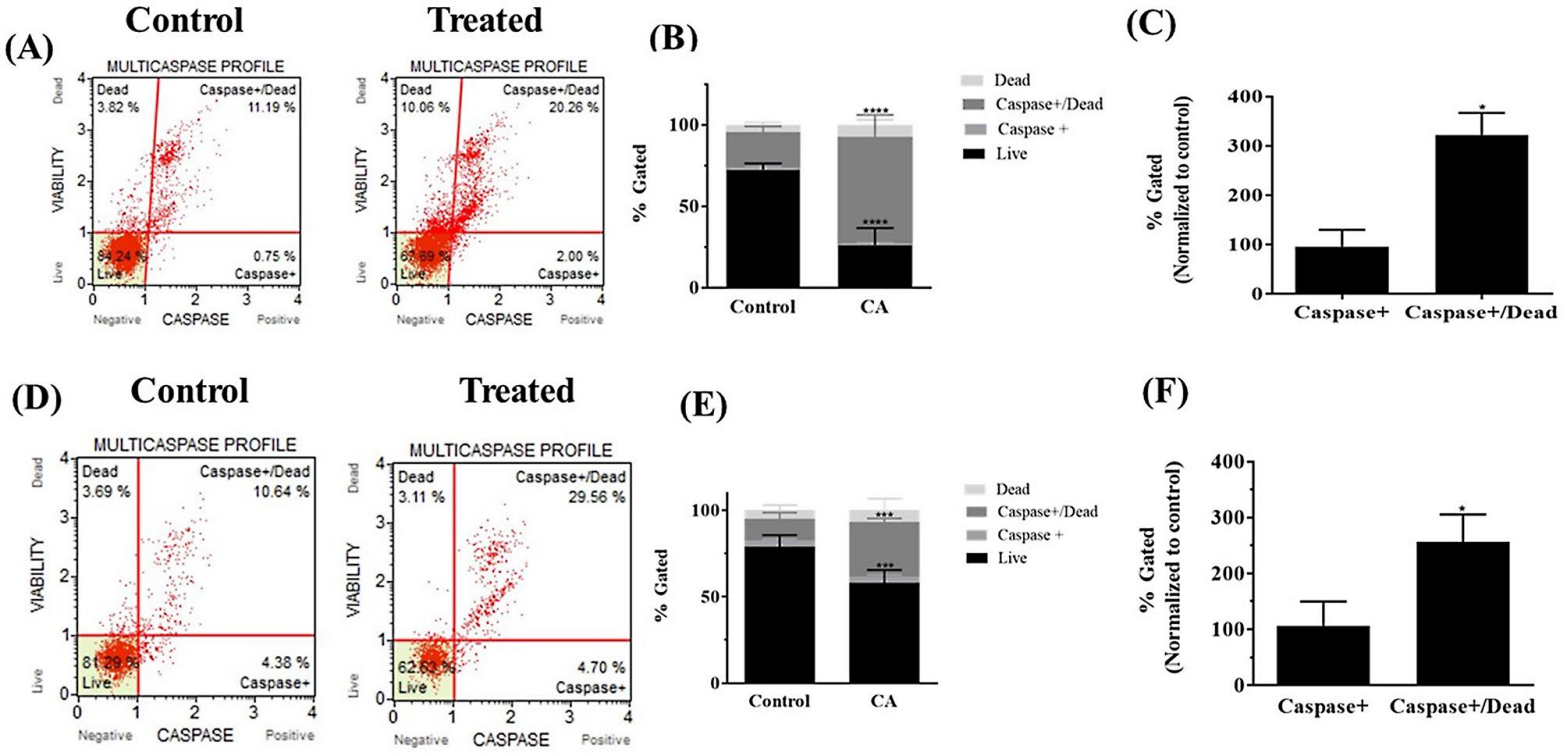
MultiCaspase were invoked in CA treated H4 and U251 cells. **(A)** Representative MultiCaspase scatter plots of H4 cells untreated and treated with 80 µM of CA. **(B)** Percent gated profile of cell population of H4 cells with Multicaspase. A statistically significant decrease in live cell population and significant increase of Caspase+/Dead population in CA treated H4 cells was observed as per two-way ANOVA with Sidak’s multiple comparisons test for both live and Caspase+/Dead population (p value < 0.0001). **(C)** On normalizing to the control group there was a statistically significant increase in the Caspase+/Dead population as per one-way ANOVA with Dunnett’s multiple comparisons test for Caspase + Dead cells (p value = 0.0204). **(D)** Representative scatter plots obtained on MultiCaspase flow cytometry analysis of U251 cells, untreated (control) and treated with 80 µM are represented. **(E)** In the U251 cells as well, a statistically significant decrease in live cell population and significant increase of Caspase+/Dead population was observed on treatment with CA as per two-way ANOVA with Sidak’s multiple comparisons test for live population (p value = 0.0002) and Caspase+/Dead population (p value = 0.0007). **(F)** On normalizing to the control group, there was a statistically significant increase in the Caspase+/Dead population as per one-way ANOVA with Dunnett’s multiple comparisons test (p value = 0.0479)

As we observed an increase in apoptotic cells in both the GBM and LGG cell lines, we proceeded further to investigate whether Multicaspase are elicited by CA in these cell lines. Activation of Multicaspase was monitored by flow cytometry using the Luminex MultiCaspase assay. Three independent experiments were performed for each of the cell lines used in the study. Representative scatter plots obtained on MultiCaspase flow cytometry analysis of H4 cells, untreated (control) and treated with 80 µM are represented, (Fig. 3A). The percent gated profile of untreated (control group) and treated cells from three independent experiments are shown by histogram, (Fig. 3B). A statistically significant decrease in live cell population and significant increase of Caspase+/Dead population in CA treated H4 cells was observed. On normalizing to the control group there was a statistically significant increase in the Caspase+/Dead population (Fig. 3C). Similar analysis was conducted for U251 cells. Representative scatter plots obtained on MultiCaspase flow cytometry analysis of untreated (control) and treated with 80 µM are represented (Fig. 3D). In the U251 cells as well, a statistically significant decrease in live cell population and significant increase of Caspase+/Dead population was observed on treatment with CA. (Fig. 3E). On normalizing to the control group, there was a statistically significant increase in the Caspase+/Dead population (Fig. 3F).

### CA does not induce mitopotential change in H4 as well as in U251 cells

Since the data from the Annexin assay as well as the multicaspase experiments suggested that the extrinsic programmed cell death pathway may be operational in H4 and U251 cells treated with CA, we wanted to explore whether intrinsic programmed cell death pathway also contributed towards the inhibition of cell viability. To this end, we performed flow cytometric analysis to detect the changes in mitochondrial membrane potential. Representative scatter plots depicting populations with mitopotential change for both H4 and U251are shown (Additional file 1: Fig, S1A and S1C). When data from three independent experiments were analyzed, no significant differences were noted between control and treatment groups (Additional file 1: Fig, S1B and S1D).

## Discussion

Treatment strategies for GBMs and LGGs with minimal side effects are essential as there is lack of such options for these malignancies of the brain. The investigation of the impact of natural products on different types of brain tumors need to be pursued as fewer side effects are expected. Recently, we have reported the potential impact of CA, an ingredient of the natural product cinnamon, on U87 (wild-type p53) glioblastoma cells. Also, we showed that CA can impact the cell viability of the GBM cells with p53 mutation (U251 cell line) as well as H4 cell line which are LGG cells [28]; but the elucidation of the molecular mechanism of action of CA on both the brain tumors GBM and LGG is essential to evaluate the cell entities and cell pathways affected. Majority of the GBMs have changes in p53; furthermore, LGGs could progress to secondary GBMs over time [23, 24]. Therefore, molecular studies with compounds that impact these brain tumors is warranted.

In the present study, we observed the impact of CA on one of the key cellular molecules, ROS; levels of which increased in both the types of gliomas. CA treatment has been shown to induce ROS in cancer cells of varied tissue origin including hepatoma [29], breast [30, 31]and renal [32]. In U251 cells, resveratrol is reported to increase ROS levels and cause cellular effects [33]. Also, curcumin is reported to target glioblastoma stem cells through induction of ROS [34]. Furthermore, molecules like Gefitinib is reported to induce apoptosis in human glioma by affecting the BAD/BAX signaling pathway including activating caspase 9/3 [35]. In fact, in the present study we observed an increase in apoptotic cells, and multicaspase were invoked in both the GBM and LGG brain tumor cell types on treatment with CA. Importantly, the pivotal Bcl-2 molecule involved in cell death/survival pathway was impacted. Notably, recently the assessment of BH3 mimetic Bcl-2 inhibitor has shown inhibition of tumor growth of glioblastoma in the in vivo studies [36]. Importantly, inhibitors of Bcl-2/Bcl-xl along with Mcl-1 inhibitors in a nanoparticle format has shown efficacy in animal model system in crossing the blood-brain barrier and suppressing the growth of glioblastoma [37]. Of note, we observed a significant impact on the levels of Bcl-2 in both the types of brain tumor cells (GBM and LGG) undertaken in the present study.

## Conclusions

Results from the current study show that CA has the ability to interfere with the glioma cell survival by induction of programmed cell death through caspases and ROS production. Additionally, the impact of CA on Bcl-2, a pivotal molecule controlling various cell pathways, suggests that CA could be a potent anticancer entity against GBM and LGG. In contrast to the clinically used chemotherapeutics like TMZ, it is anticipated that CA will have minimal adverse effects. If the current findings can be translated in vivo, CA could also impede the progression of LGG to aggressive GBM.

### Limitations and future research

One of the limitations of the current study is that CA was tested alone. Combinatorial studies with CA and the currently clinically used chemotherapeutic TMZ, should be investigated to assess potential synergy which would minimize adverse side effects of TMZ. As stem-like cells are the contributors for cancer recurrence, future investigations should be directed towards analyzing the effects of CA on stem-like cells from gliomas. Additionally, in vivo studies with CA in appropriate glioblastoma xenograft models are needed to determine its translational potential.

## Electronic supplementary material

Below is the link to the electronic supplementary material.

Supplementary Material 1

## Data Availability

No datasets were generated or analysed during the current study.
