# Supplementary material for: Cinnamaldehyde impacts key cellular signaling pathways for induction of programmed cell death in high-grade and low-grade human glioma cells
Source: BMC Res Notes. 2025 Jan 20;18:23. doi: 10.1186/s13104-025-07092-8 (PMC11744947; doi:10.1186/s13104-025-07092-8)
Supplement: Supplementary file 1 — Supplementary Material 1 [file 13104_2025_7092_MOESM1_ESM.docx]

**Supplementary Information**

**Additional file 1: Figure S1**

**
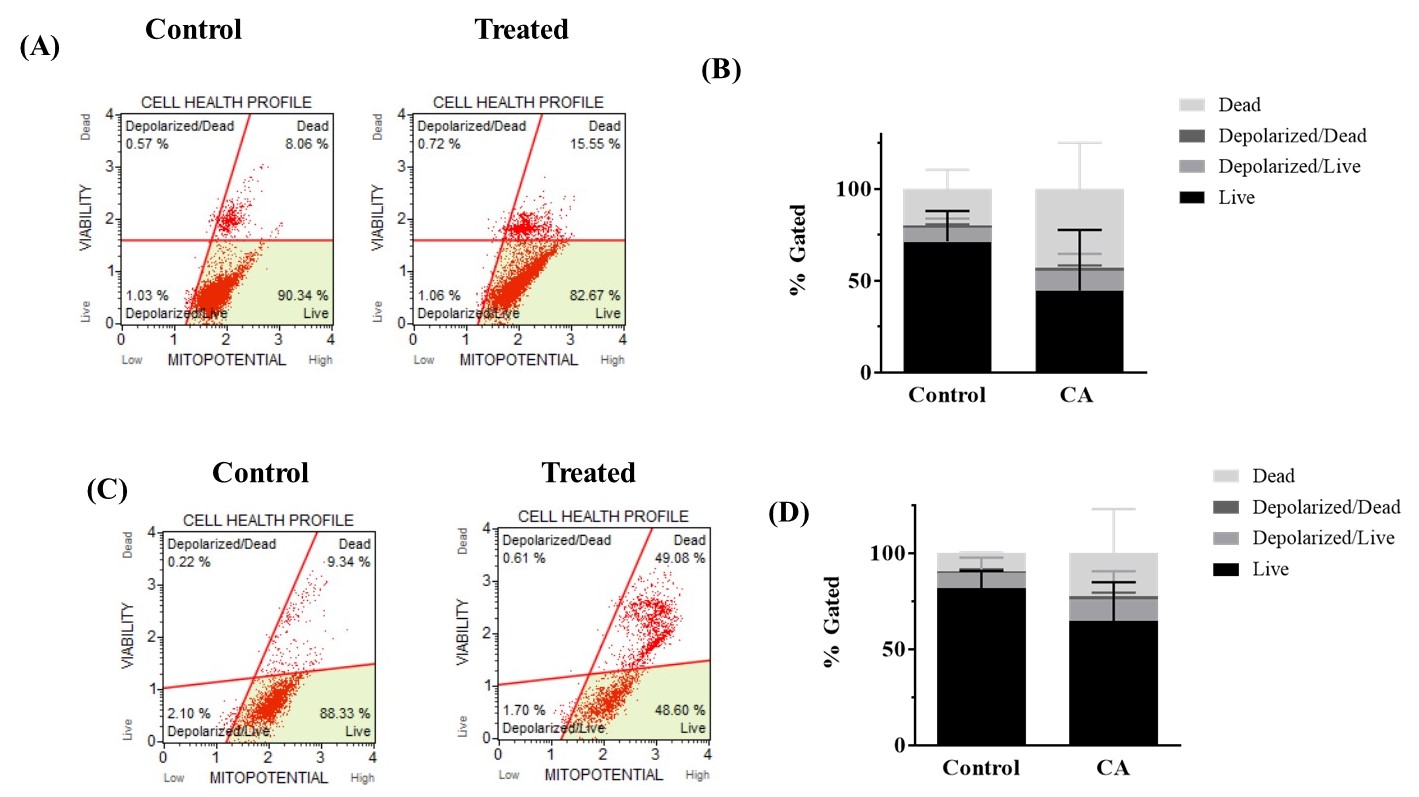
**

**Analysis of mitopotential change in U251 and H4 cells treated with CA**. **(A)** Representative scatter plots of mitopotential change in H4 cells treated with CA. **(B)** Percent gated H4 cell populations are depicted. **(C)** Representative scatter plots of change in mitopotential in U251 cells treated with CA. **(D)** Quantitation of the various U251 cell populations with mitopotential change. Data is from three independent experiments. In both cases, H4 and U251, no statistically significant differences were observed in the treated groups in comparison to the control groups.
